# Collaborative planning approach to inform the implementation of a healthcare manager intervention for hispanics with serious mental illness: a study protocol

**DOI:** 10.1186/1748-5908-6-80

**Published:** 2011-07-26

**Authors:** Leopoldo J Cabassa, Benjamin Druss, Yuanjia Wang, Roberto Lewis-Fernández

**Affiliations:** 1 New York State Center of Excellence for Cultural Competence, New York State Psychiatric Institute, New York, USA; 2 Department of Psychiatry, College of Physicians & Surgeons, Columbia University, New York, USA; 3 Rollins School of Public Health, Emory University, Atlanta, USA; 4 Mailman School of Public Health, Columbia University, New York, USA

## Abstract

**Background:**

This study describes a collaborative planning approach that blends principles of community-based participatory research (CBPR) and intervention mapping to modify a healthcare manager intervention to a new patient population and provider group and to assess the feasibility and acceptability of this modified intervention to improve the physical health of Hispanics with serious mental illness (SMI) and at risk for cardiovascular disease (CVD).

**Methods:**

The proposed study uses a multiphase approach that applies CBPR principles and intervention-mapping steps--an intervention-planning approach--to move from intervention planning to pilot testing. In phase I, a community advisory board composed of researchers and stakeholders will be assembled to learn and review the intervention and make initial modifications. Phase II uses a combination of qualitative methods--patient focus groups and stakeholder interviews--to ensure that the modifications are acceptable to all stakeholders. Phase III uses results from phase II to further modify the intervention, develop an implementation plan, and train two care managers on the modified intervention. Phase IV consists of a 12-month open pilot study (N = 30) to assess the feasibility and acceptability of the modified intervention and explore its initial effects. Lastly, phase V consists of analysis of pilot study data and preparation for future funding to develop a more rigorous evaluation of the modified intervention.

**Discussion:**

The proposed study is one of the few projects to date to focus on improving the physical health of Hispanics with SMI and at risk for CVD by using a collaborative planning approach to enhance the transportability and use of a promising healthcare manager intervention. This study illustrates how blending health-disparities research and implementation science can help reduce the disproportionate burden of medical illness in a vulnerable population.

## Introduction

This manuscript describes an innovative, collaborative intervention-planning approach that capitalizes on both researchers' and stakeholders' knowledge and skills to inform pre-implementation work to transport a promising healthcare manager intervention to a new patient population and provider group. Our approach builds upon the growing interest in using community-based participatory research (CBPR) as a translational strategy to bridge the gap between research and practice in underserved diverse communities in the United States in order to reduce health inequities [1]. We combined principles from CBPR (*e.g.*, capacity building, ownership) and an intervention mapping (IM)--a step-by-step systematic framework for intervention planning, implementation, and evaluation [2]--to modify and assess the feasibility and acceptability of an existing care manager (CM) intervention to improve the physical health of Hispanics with serious mental illness (SMI; *e.g.*, schizophrenia) and at risk for cardiovascular disease (CVD).

## Background

Compared to non-Hispanic whites with SMI, Hispanics with SMI have higher rates of obesity [3], diabetes [4,5], and other metabolic risk factors[6,7], placing them at elevated risk for CVD. These health needs are exacerbated by the fact that Hispanics with SMI and at risk for CVD are less likely to engage and receive high-quality medical care, even against the backdrop of the poor-quality care received by people with SMI [8]. Despite higher risk and disparities in care, limited attention has been paid to the process of making interventions shown to improve the physical healthcare of people with SMI, such as CM interventions, culturally relevant to Hispanics with SMI. Cultural modifications are important because unique sociocultural factors (*e.g.*, cultural norms) influence access and quality of care, and the provision of culturally sensitive care increases engagement and treatment retention [9]. Provider-level modifications are also needed because many public mental health systems--the main source of healthcare for Hispanics with SMI [10]--lack sufficient numbers of registered nurses (RNs) [11,12], the provider group for whom these interventions have been designed. The absence of an established approach for systematically conducting intervention modifications and pre-implementation work in order to expand the intervention's use with a new patient population and provider group without compromising its effectiveness constitutes an implementation research gap. This study uses a collaborative planning approach that blends principles of CBPR and IM to modify and assess the feasibility and acceptability of an existing CM intervention to improve the physical health of Hispanics with SMI and at risk for CVD.

### Study aims

The specific aims of this study are as follows:

1. Use a collaborative intervention-planning framework to conduct intervention modifications.

2. Pretest intervention methods and materials with Hispanics with SMI and at risk for CVD.

3. Identify stakeholders' views of factors impacting the acceptability and sustainability of the modified intervention.

4. Pilot test the feasibility and acceptability of the modified intervention and initially explore its effect.

### Care manager interventions

CM interventions are a cornerstone of initiatives to improve the health of people with multiple and complex physical and mental health conditions [13-15]. CM interventions improve receipt of primary care services, social functioning, independent living skills, and the quality of life of people with SMI and medical conditions linked to CVD (*e.g.*, hypertension) [16,17]. The Primary Care, Access Referral and Evaluation (PCARE)[18], a CM intervention developed by one of the authors (BGD), focuses on improving patient activation and the coordination of medical care between outpatient mental health clinics and primary care. In a recent randomized controlled trial, the intervention was found to double the rate of receipt of preventive medical care and to improve the quality of cardiometabolic care and mental-health-related quality of life among adults with SMI [18]. CMs do not provide direct medical services; instead, they work individually with patients in the mental health clinic to provide education and activation and to connect patients to primary care, as well as coordinate their medical care between mental health and primary care providers.

### Adapting the intervention to a new patient population and provider group

Adapting any healthcare intervention to a new population requires consideration of both sociocultural and system-level factors that can affect its uptake. Adapting PCARE to Hispanics provides an example of attention to both patient and system-level considerations.

First, unique sociocultural factors that impact patient activation and care coordination--core elements of CM interventions--need to be examined and incorporated into the intervention to ensure its acceptability, feasibility, and effectiveness in the new population. Sociocultural adaptations involve surface- and deep-level modifications [19]. Surface modifications entail matching intervention materials, messages, and content to the observable characteristics of the new patient population in order to enhance the intervention's "face validity." Examples of surface elements include delivering the program in the patient's dominant language (*e.g.*, Spanish) to reduce language barriers and drafting all patient educational materials at the appropriate reading level (*e.g.*, fourth grade) to enhance health literacy. Surface adaptations are a prerequisite, but they may not be sufficient to attain cultural sensitivity; thus, deep-level modifications are required.

Deep-level modifications entail identifying and incorporating into the intervention patients' cultural values, understandings, and preferences that impact core intervention elements [19]. The qualitative research conducted by our team with six behavioral health organizations in the Northern Manhattan communities of New York City serving Hispanics and African Americans with SMI illustrates how sociocultural factors impact patient activation and care coordination [20]. The medical encounter itself, for example, is shaped by cultural factors. We found that many Hispanics with SMI, who are disenfranchised due to their mental illness and minority status, feel that it is inappropriate and disrespectful to directly question their doctors' advice. CMs need to recognize this deference to authority and adapt their patient-activation techniques accordingly, such as by modeling appropriate interactions with providers and helping patients formulate a list of questions before their medical visit [21]. Similarly, we found that body image varies across cultures, with some Hispanic patients favoring a fuller body ideal [22,23]. Unaddressed, this can lead to the perception among Hispanics that medical recommendations that focus on thinness may be culturally insensitive. CMs can deal with this by assessing patients' explanatory models of illness and using this information to present medical goals congruent with patients' views (*e.g.*, focusing on health rather than thinness). Also, in contrast with many white consumers, most Hispanics with SMI live with their families [24-26], who are involved in their medical care. This suggests that family members should be included in medical care decisions, as appropriate, to enhance this source of support. These examples illustrate the kind of deep modifications needed to ensure that the intervention is congruent with patients' culture, without diluting its effectiveness [19,27].

Second, whereas PCARE was developed for use with RNs, these practitioners were in short supply both in our study site and in other sites where Hispanics with SMI receive their care [11]. In our study site, for example, social workers (SWs) outnumber RNs 2.5:1. Since CMs provide no direct medical care, our plan to modify the intervention to SWs is appropriate and essential to increase applicability to the target population. SWs are a natural fit for the CM role because they [1] deliver approximately 60% of the mental healthcare in the United States [28], particularly in the public system; [2] can bill for care management functions at our study site; and [3] work closely with minority communities. Their expertise in counseling and systems navigation match the skills required for effective care management. Multiple studies have demonstrated the effectiveness of SWs as CMs across a range of mental and physical illnesses [29-32]. To modify PCARE for SWs requires that they receive extra training on how to use existing guidelines to monitor and coordinate the management of the physical health needs of people with SMI [33], deliver appropriate physical health information to patients, and coordinate medical care between mental health and primary care providers. SWs will also require more intense clinical training on how to manage the medical needs of patients with complex medical problems. Due to medical and legal regulations, SWs will need more structured and regular supervision and involvement from existing RNs and/or primary care providers.

Lastly, the addition of this intervention to the study site requires attention to providers' attitudes and expectations about the intervention and identifying what resources are needed to accommodate the intervention into routine practice. More generally, adapting interventions to vulnerable ethnic subpopulations calls for close attention to not only cultural factors but also to these provider and system-level considerations.

### Collaborative planning approach

**Figure 1:**
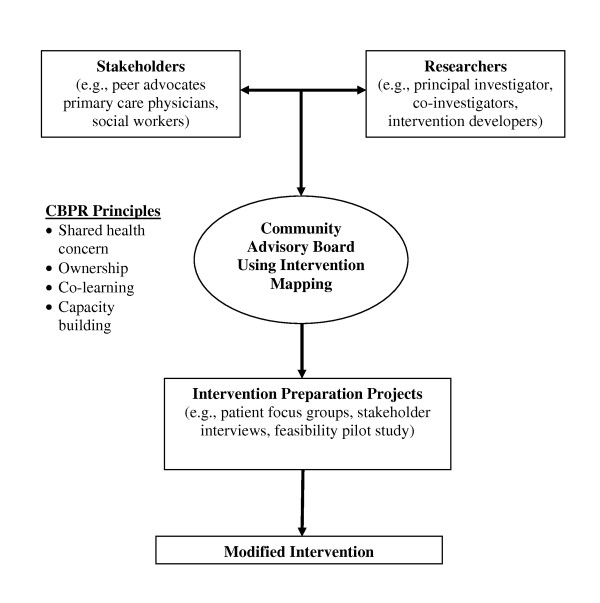
**Collaborative planning approach: blending community based participatory research (CBPR) and intervention mapping**.

Figure 1 presents the collaborative framework for this study, which blends CBPR principles and IM procedures to plan and implement these cultural and system-level modifications. CBPR in intervention research can be conceptualized as a translational strategy that focuses on co- learning, engendering ownership, and two-way capacity building among all partners to address a shared health concern and develop sustainable solutions [1]. CBPR differs from other knowledge translational approaches for its emphasis on community members' participation as equal contributors in creating knowledge and skills that inform the implementation process [34-36]. According to Wallerstein and Duran [1], CBPR enhances the potential of implementation sciences in diverse communities through the use of strategies that "redress power imbalances, facilitate mutual benefits among community and academic partners, and promote reciprocal knowledge translation incorporating community theories into the research" (p. S1). CBPR principles in our framework include a focus on a shared health concern identified by our community partners, formation of a community advisory board (CAB) of community and academic partners to maximize the relevance of the intervention and incorporate community knowledge and wisdom into the implementation process, and involving partners in all modification steps to enhance their capacity for adopting and using the intervention.

Although CBPR approaches have been used to modify interventions to address health disparities [37,38], the procedures for how to carry this out remain unspecified and difficult to replicate. Clear guidelines and procedures are needed to help community members and researchers move through a collaborative process to plan, implement, and evaluate health interventions (see Belansky and colleagues [39] for a recent example). Our collaborative framework addresses this limitation by using IM in a CBPR partnership (see Additional file 1) [40]. IM "road maps" planners' moves from theory to practice. It uses core processes (*e.g.*, brainstorming, literature reviews, data collection) and visual tools, such as logic models and matrices of performance objectives, to clarify intervention goals and how to attain intended outcomes [40]. Similar to CBPR, IM relies on bringing together a planning team or linkage system that includes researchers, program participants, potential program users, and other stakeholders to inform intervention planning, implementation, and evaluation. Most IM projects to date have tended to be driven by researchers and/or public health interventionists [40]. More recently, several teams have begun to incorporate community members' participation and leadership into the process [39,41]. Our approach will build upon these recent examples.

The six steps of IM that inform intervention modifications are [1] problem analysis, [2] review of intervention objectives and theoretical foundations, [3] modification of intervention methods and strategies, [4] development of a revised intervention, [5] development of an implementation plan, and [6] evaluation [42]. Each step includes specific tasks leading to a product (*e.g.*, logic model) that is the basis for the next step. The result is an "intervention map" consisting of tables, logic models, and written plans that guide the modification, implementation, and evaluation of the intervention [2]. We chose IM over other adaptation models [43-45] because [1] it relies on group members' input throughout the entire process; [2] at each step the goals of the intervention are emphasized, engendering participation of CAB members with divergent backgrounds; and [3] it develops an implementation plan, composed of objectives and strategies, that supports the adoption and use of the intervention in a new context. In sum, this collaborative framework provides a structured and participatory platform for directing intervention modifications and pre-implementation work.

## Methods

### Setting

The Washington Heights Community Service (WHCS) of the New York State Psychiatric Institute will serve as the study site and community partner. WHCS is a state-run public mental health clinic located in the largest Hispanic community in New York state; its catchment area is approximately 74% Hispanic, mostly first-generation Dominican. About one-third of residents live in poverty, 31% lack health insurance, and 25% lack a regular medical home [46]. WHCS serves those members of the community that are most disenfranchised as a result of SMI. In 2008, WHCS had 1,030 patients, of whom 75% were Hispanics, 40% were male, and 90% had schizophrenia-spectrum or bipolar disorder. A study of 69 WHCS patients (78% Hispanic) revealed their poor health status [3]. Although 67% had had a physical exam over the past year, 89% of women and 59% of men were overweight/obese (body mass index > 25 kg/m^2^). Many had elevated rates of CVD risk factors: hypertension (29%), hyperlipidemia (22%), and diabetes (17%). The proposed modified CM program aims to help address these needs.

### Study design overview

The project will use a multiphase approach (see Table 1) that applies IM steps to move from intervention planning to pilot testing. Study phases are described below.

**Table 1:** Overview of study phases

Phase	Activities	Intervention mapping steps
**I**	Assemble CAB, review PCARE, make initial surface modifications	1-3
**II**	Conduct four patient focus groups and 20 stakeholder interviews to ensure that modifications are acceptable to all stakeholders	3
**III**	Use results from phase II to modify PCARE, develop implementation plan, train a social worker, finalize pilot protocol	4-5
**IV**	Conduct 12-month open pilot study (N = 30) to assess feasibility and acceptability and explore initial effects	6
**V**	Analyze pilot study data and prepare manuscripts and future grant proposals	6

CAB = community advisory board; PCARE = primary care, access referral and evaluation.

### Phase I

During phase I, we will assemble the CAB composed of one primary care physician, one WHCS social worker, two consumer advocates (*e.g.*, former consumers), one clinic administrator, and the research team to inform all phases of the project. Use of a CAB is an established CBPR strategy for modifying interventions as it helps frame and monitor the progress of the project and provides guidance on cultural values and practices as well as community assets that deepen the contextualization of the intervention [47]. The CAB will meet monthly during phase I and every three months through phases II-V. IM steps 1-3 will be completed in this phase. IM step 1 (problem analysis) entails deepening CAB members' understanding of the physical health needs of Hispanics with SMI by reviewing existing literature, discussing results of a pilot qualitative study on the physical health needs of minorities with SMI [20], and discussing the capacity of the study site and other community agencies to address these needs. We will also discuss how to address possible provider-level barriers, such as primary care doctors' stigma and reluctance to work with patients with SMIs and the lack of communication between primary care and mental health providers. IM step 2 (review of intervention objectives and theoretical foundations) involves learning and reviewing intervention components. Intervention objective tables will be constructed to specify the links between each intervention objective and the methods used to achieve it. The CAB will determine whether objectives and determinants of these objectives need to be added or deleted without compromising intervention effectiveness and whether the intervention methods are practical and appropriate for the new patient population and provider group to achieve desired outcomes. In IM step 3 (modification of intervention methods and strategies), we will use results from patient focus groups (FGs) and stakeholder interviews (see below) to guide initial modifications. We expect to make several modifications at this stage, such as translating PCARE materials into Spanish, developing a community resource guide to the Washington Heights neighborhood, adding explanatory model questions about consumers' physical health problems to the assessment tool, and developing strategies to involve family members in CM sessions.

Another likely modification is to supplement the intervention with culturally and linguistically appropriate patient education tools, such as health-related *fotonovelas *[48,49]. *Fotonovelas *use posed photographs, text bubbles with simple text, and soap opera narratives to engage patients and raise their knowledge about specific medical conditions (*e.g.*, diabetes) and lifestyle changes. At the end of phase 1, we will have an initial modified version of PCARE.

### Phase II

During this phase we will continue IM step 3 and use qualitative methods (patient FGs and stakeholder interviews) to pretest our initial modifications and ensure their feasibility and acceptability.

### Patient focus groups

Four patient FGs conducted at the WHCS will be used to pretest the intervention methods and materials. Each FG will last 90 minutes and will consist of 8-10 patients who meet inclusion criteria for participation in the intervention. We will recruit up to 40 adult Hispanic patients with SMI and at risk for CVD at WHCS through flyers, word of mouth, and provider/peer referrals. Eligible patients are active patients at WHCS who are 18 years of age or older; self-identify as Hispanic; speak English or Spanish; and have chart diagnoses of schizophrenia, schizoaffective disorder, or bipolar disorder and have at least one CVD risk factor (body mass indent >  25 kg/m^2^], diabetes mellitus, hypertension, or hyperlipidemia). CVD risk factors are mandated by the New York State Office of Mental Health (OMH) to be part of patient medical records. We will exclude patients who are in need of detoxification; are at acute risk of suicide or homicide; fail a capacity-to-consent questionnaire [50]; or are cognitively impaired, as detected on the Mini-Cog Examination, which does not require English fluency or formal education [51]. To accommodate patients' language preferences, FGs will be conducted in Spanish or English. LJC will facilitate all FGs. FGs will be audiotaped and transcribed. A FG guide of six to eight open-ended questions developed with input from the CAB and implementation research consultants will be used to elicit patients' views about intervention materials and methods. Questions will explore patients' views about the acceptability, feasibility, and cultural appropriateness--particularly among the main Hispanic subgroups in our study site (Dominicans and Mexicans)--of the intervention, including suggestions for improvement.

### Stakeholder interviews

Twenty stakeholder semi-structured qualitative interviews will be conducted to examine the acceptability and sustainability of the modified intervention in a public mental health system. A purposive sample of stakeholders will be obtained, including five mental health clinicians, five primary care physicians, five consumer advocates, and five administrators. The aims of these interviews are to identify factors that facilitate or impede the acceptability and sustainability of the intervention and identify strategies to maximize its acceptability and sustainability (*e.g.*, adding a CM training curriculum for SWs). Interviews will last 60 minutes and will be conducted by LJC or a trained research assistant (RA) in person or via telephone to accommodate participants' availability. The option of telephone interviews enables us to reach stakeholders across New York state. Interviews will be audiotaped and transcribed. Participants will be stakeholders who are at least part-time employees at their organizations and consumer advocates who are involved in the OMH advisory committees. Recruitment strategies include presentations at OMH meetings focusing on physical/mental health integration and referrals from the Director of Research at the OMH. An interview guide will be developed with input from the CAB and the implementation and qualitative consultants. Questions will explore factors and barriers that influence an intervention's acceptability and sustainability, such as characteristics of the intervention (*e.g.*, complexity, cost), the organization's financial resources and openness to change, staff's capacity, knowledge, self-efficacy, and competing demands [52].

### Phase III

IM steps 4 and 5 will be completed in this phase in order to further modify the intervention, develop an implementation plan, and train two SWs from the study site on the modified intervention. IM step 4 (development of revised intervention) will entail using phase II findings to reexamine, and if necessary reconstruct, program objective tables developed in phase 1. This process will include CAB review of salient themes that emerged from phase II FGs and interviews, refinement of the program's logic model of change, and discussions about revising objectives, methods, and/or strategies. To ensure that modifications do not compromise intervention core elements, revisions will be presented and discussed with the second author (BD), the PCARE developer. The goal of this step is for the CAB to determine if intervention objectives and methods should be unchanged, deleted, revised, or added. Table 2 presents examples of possible modifications.

**Table 2:** Examples of possible PCARE modifications

Sociocultural modifications		Provider-level modifications
Surface level	Deep level	
• Translate intervention materials into Spanish • Use bilingual care managers • Adapt community resource guide to Washington Heights	• Model interactions with medical providers and help patients formulate questions before medical visits • Reframe lifestyle change in terms of health, not thinness • Use health-related *fotonovelas* • Involve family members in decision making, as appropriate	• Add training session on CVD risk factors for SWs • Add training module for SWs on how to work effectively with PCPs • Distribute pocket cards to CMs and PCPs summarizing CVD risk factors

PCARE = primary care, access referral and evaluation; CVD = cardiovascular disease; SW = social worker; PCP = primary care provider; CM = care manager.

During IM step 5 (development of implementation plan), the CAB will formulate specific implementation objective tables and methods. In this step, the CAB will (a) discuss themes that emerged from phase II interviews and FGs regarding intervention acceptability, feasibility, and sustainability; (b) identify existing resources (*e.g.*, connections with primary care clinics) that facilitate intervention implementation; and (c) develop a performance objective table that links each implementation objective to the methods used to accomplish it. The goal is to write a detailed plan of what needs to be done to ensure the intervention is delivered at acceptable levels of fidelity and completeness. The plan will include CM tracking forms to log frequency, number, and types of patients' contacts and clinical services. These will serve as fidelity indicators for structural and clinical elements of the intervention [53].

Once the revised intervention is finalized, two SWs will be trained. Training will consist of four 90-minute education sessions that combine didactic presentations and interactive hands-on activities, such as role-playing, teach-back, and practice exercises. The training will be taught by intervention developers and other project consultants who are experts in primary care and cardiovascular care. The sessions will be based on the intervention materials, the American Heart Association guidelines for managing CVD risk factors [54], and the New York City Department of Health and Mental Hygiene continuing education modules that review existing guidelines for monitoring the physical health of adults with SMI [33]. Training topics include ascertainment of CVD risk factors; monitoring these risk factors in an SMI population; learning how to correctly obtain simple body composition measures (*e.g.*, weight, height), blood pressure assessment, and a basic medical history; developing patient education skills to boost patient recall and comprehension using teach-back techniques [55]; basics of motivational interviewing techniques; use of action plans [56]; and coordination with local medical care services. We will also develop and train SWs on how to follow specific clinical protocols to manage possible medical emergencies (*e.g.*, myocardial infarction, diabetic ketoacidosis) that require immediate attention by a medical provider (*e.g.*, clinic's nurse, emergency department). Lastly, during the pilot trial (see below) the two SWs will receive supervision every two weeks from the project primary care consultant, an experienced family physician, who will also provide clinical backup in case of medical emergencies. During these meetings, SWs will review patients' initial assessment and stated health goals and identify additional health needs, potential barriers to care, and strategies for ensuring receipt of services. An individualized patient report will be generated that identifies key medical issues, providers, and contacts, as well as short- and long-term goals. A copy of this report will be given to the patient, and if the patient consents, added to his/her medical records and given to his/her primary care provider.

### Phase IV

This phase will complete IM step 6 (evaluation) with a single group, pre-post, 12-month open pilot study (N = 30) carried out at WHCS. Two SW CMs will deliver the modified intervention, each carrying a caseload of 15 patients. Thirty adult Hispanic patients with SMI will be recruited. Inclusion and exclusion criteria are identical to those in phase IV. Based on the PCARE study recruitment rate of 69%, we will screen at least 45 patients to achieve our recruitment goal. A bilingual RA will recruit patients at WHCS through flyers, canvassing of waiting rooms, word of mouth, and provider referrals. We have allocated 18 months to this phase, which provides a six-month cushion for unanticipated problems with recruitment and attrition. Descriptions of the proposed measures for this study are presented in Table 3. As part of the planning process for this phase, the CAB will assess the appropriateness of these outcome measures for the intended patient population and, if necessary, identify other culturally relevant measures for key constructs (*e.g.*, acculturation, health literacy, illness perceptions).

**Table 3:** Description of measures

Construct	Measure description
Feasibility	Study recruitment, assessment completion, and treatment attendance rates
Acceptability	CSQ and postintervention focus groups. The CSQ is an eight-item questionnaire scored on a four-point Likert-type scale. Scores range from 8 to 32, with higher score indicating higher satisfaction [68]. The CSQ is available in English and Spanish [69] and has excellent internal consistency in Hispanics [70].
Patient activation	PAM-13, [71] a 13-item scale that assesses patients' knowledge, skills, and confidence about self-management. Scores range from 0 (no activation) to 100 (high activation). PAM-13 has strong psychometric properties, including content and construct validity [71], has been tested across a range of chronic illnesses [72,73], and is available in English and Spanish.
Receipt of preventive primary care	PCARE study measure drawn from the USPSTF guidelines [74]. A total of 23 indicators are examined across four domains: (1) physical examinations, (2) screening tests, (3) vaccinations, and (4) education. Scored based on patients' medical records and self-report at baseline, three months, and six months. An aggregate preventive services score calculates the proportion of appropriate services obtained [18].
Health and mental health-related quality of life	SF-12 [75], a self-report measure available in English and Spanish and validated among adults with SMI that generates two summary scores for physical and mental health-related quality of life [76]. Summary scores range from 0 to 100, with higher scores reflecting better health.
Covariates	Demographics: *e.g.*, race/ethnicity, age, gender, education, and marital status Acculturation: nativity, language dominance, time in the United States, age of migration; the Bidimensional Acculturation Scale [77], a 24-item self-report measure that uses separate four-point scales to tap acculturation-related changes in two languages. Barriers to medical care: a list of 11 common factors that may prevent patients from seeking medical care [78,79]. Comorbid medical conditions: a list of 17 common medical conditions by patient self-report.

CSQ = Client Satisfaction Questionnaire; PAM-13 = Patient Activation Measure; PCARE = primary care, access and referral; USPSTF = U.S. Preventive Services Task Force; SF-12 = Short Form Health Survey; SMI = serious mental illness.

The primary outcomes of the pilot study are feasibility and acceptability. Two post-intervention FGs will be conducted with study participants to explore their reactions to the intervention. Each FG will be composed of 8-10 patients and follow the FG methods described in phase II. Although this is not an efficacy trial, we would be remiss in not taking this opportunity to initially explore the intervention's effect, as measured by patient activation, receipt of preventive primary care, and physical- and mental-health-related quality of life. Covariates include demographics, acculturation, barriers to care, and comorbid medical conditions. The RA will assess patients at baseline, 6 months, and 12 months. Self-report measures and medical chart abstractions will be obtained. Patients will be asked for consent to access medical records at WHCS and their primary care clinic. The RA will conduct these abstractions under LJC's supervision.

### Phase V

This final phase will consist of analysis of pilot study data, incorporation of pilot findings into the refinement of the modified intervention, and preparation for future funding to develop a more rigorous evaluation of the intervention. A concurrent triangulation approach [57] will be used to integrate qualitative and quantitative data generated from this pilot. A set of questions will guide this data integration process to identify themes, patterns, and conflicts: What patterns emerge from each set of findings? Do they converge? If not, what are their discrepancies and what additional data and analysis are needed to understand these discrepancies? All study results will be presented and discussed with the CAB to inform next steps and future plans.

### Data analysis

Quantitative data entry and analysis will utilize SAS version 9.1.3 (SAS Institute, Inc., Cary, NC, USA). ATLAS.ti (ATLAS.ti Scientific Software Development GmBH, Berlin, Germany), a qualitative data management software [58], will be used to manage and analyze all qualitative data.

### Quantitative analysis

All tests will be two-sided and performed at significance level α = 0.05.

### Preliminary analyses

Preliminary analyses will consist of examining distributions of all baseline variables, identifying outliers, and calculating descriptive statistics. For longitudinal data, we will examine the distributions and calculate descriptive statistics for variables relevant to study aims at each time point. Proper transformation will be considered to meet assumptions of parametric models. Graphics will be used to explore bivariate associations, especially patterns over time for variables of interest.

### Accounting for missing data in the analysis

If a patient drops out of the study, we will record the stated reasons for dropout and make all efforts to obtain a final assessment. Failing this, the end-of-treatment assessment will be the patient's last scheduled assessment. The analysis of acceptability as measured by the Client Satisfaction Questionnaire (CSQ) will be based on a multilevel model with subject-specific random intercepts. For analyses that include missing outcomes, PROC MIXED in SAS allows for continuous data that may be missing for some patients due to lack of completion of assessments. Measurement at each time point on each patient contributes to the analysis. Thus, complete outcome information for all patients at all sessions is not needed. For analyses that include missing covariates, we will use multiple imputation to account for missing data. The inferences from incomplete data are valid, provided the missing data are missing at random [59]. Unfortunately, this assumption is untestable in most medical research. When we suspect this assumption does not hold, we will assume a model for the missing mechanism that depends on the unobserved outcome value and do the analysis incorporating the assumed model and conduct sensitivity analysis [60-62].

### Primary analysis

To evaluate feasibility, we will estimate recruitment rate, assessment completion rate, and treatment attendance rate by sample proportions and provide their standard errors based on binomial distribution. We will evaluate acceptability by assessing patient satisfaction (CSQ scores) repeatedly over time (baseline, 6 months, and 12 months). We will analyze improvement in patient satisfaction by a linear mixed-effects model with random subject-specific intercepts to handle correlation between repeated measurements of the outcomes. We will include time as predictor of interest. Significance of improvement in satisfaction over time will be assessed by testing whether the slope of time is zero.

### Exploratory analysis

To explore the initial effect of the CM intervention, we will assess average level of patient activation, rates of guideline-concordant preventive primary care use, and physical- and mental-health-related quality of life. We will provide confidence intervals for these estimates.

### Precision analysis

Since this is a pilot study for a future large randomized trial, we did not conduct a power analysis in accordance with recommendations in the new National Institutes of Health guidelines http://grants.nih.gov/grants/guide/pa-files/PAR-09-173.html and Kraemer and Kupfer [63]. Instead, we conducted precision analysis for the main study outcomes--feasibility and acceptability--by computing margin of error of an estimated rate to assess the precision that can be achieved by the proposed sample size. Margin of error is half the width of a confidence interval of an estimator. The smaller the margin of error, the tighter the confidence interval and the more precision we achieve for an estimator. We used the original PCARE [18] study treatment and completion assessment rates to estimate standard errors and margins of errors. PCARE's observed treatment completion rate was 0.68, with a sample size of 30, the standard error was 0.085 and the margin of error was 0.167. PCARE's observed assessment completion was 0.78 at 6 months and 0.69 at 12 months, the corresponding standard error is 0.076 and 0.084, respectively, and the margin of error is 0.15 and 0.17, respectively. For the CSQ scores (acceptability measure), when the standard deviation of the improvement of CSQ is 1, 2, or 4 points, the corresponding margin of error for estimating mean improvement is 0.18, 0.36, and 0.72, respectively. In all, our precision analysis indicates we have small margin of errors for each of our main study outcomes.

### Qualitative analysis

All FGs and stakeholder interviews will be transcribed. The RA will review all transcripts while listening to the recordings to fix errors. Clean transcripts will be entered into ATLAS.ti. A grounded theory approach will inform the analysis [64-66]. LJC and the RA will independently read all transcripts and develop an open coding schema based on *a priori *and emergent themes. Each code condenses the data into analyzable units, ranging from a phrase to paragraphs. They will then meet with the project qualitative consultant to present their coding schemes, discuss emergent themes, refine codes, and develop a final codebook that consists of a list of categories and topics. We will use ATLAS.ti to mark instances where each code occurs in the data. To establish coding reliability, both LJC and the RA will independently code up to 10% of the transcripts. They will meet weekly during this process to review definitions and assignment of codes and resolve differences through consensus by checking the segment of transcript in question. Successive coding sessions will continue until an agreement of 85% or greater in codes applied is reached. Once this rate is maintained, a single rater will code the rest of the data. Additional analyses will include identification of subthemes and descriptions of the range and salience of themes. Established procedures to enhance the trustworthiness of our analysis will be used, including analyses of negative cases that do not fit our coding scheme, development of an audit trail documenting analytical decisions, and member-checking presentations to the CAB [67]. Qualitative results will be used to inform both surface- and deep-level modifications to enhance the cultural relevance of the intervention. Moreover, results from stakeholder interviews will enable us to identify implementation barriers as well as implementation strategies that need to be taken into consideration as we develop the implementation plan in phase III. In sum, the qualitative analysis is intended to provide insights into contextual factors that can inform intervention planning and implementation.

## Discussion

This study will contribute to the advancement of implementation science by developing a collaborative approach that blends CBPR principles and IM procedures for overcoming barriers to the modification, pre-implementation, and use of evidence-based approaches in real-world settings. This study's strengths and innovations include [1] the testing of an innovative strategy for modifying interventions to vulnerable ethnic subgroups; [2] the preparation of an intervention for Hispanics with SMI that is urgently needed to address physical health disparities that have received limited attention; [3] the modification of a promising intervention to a different provider group that can improve access to preventive primary care, reduce risk for CVD, and reduce premature mortality among Hispanics with SMI; and [4] the engagement of stakeholders in a collaborative effort to enhance the transportation of evidence-based interventions to underserved racial and ethnic minorities with SMI.

Several study limitation must be noted. This study will be conducted in one public outpatient mental health clinic located in northern Manhattan in New York City, thus limiting its generalizability to other urban areas in the United States. Cultural modifications to the intervention will focus on Hispanic adults with SMI, predominantly of Dominican or Mexican descent. Future studies are needed to examine whether these modifications are appropriate with other Hispanic groups. The open trial design of our pilot study will not permit us to test the efficacy of the modified healthcare manager intervention or to identify mechanisms of change. We plan to use the results of the pilot study to inform the design of a more rigorous effectiveness trial of the modified intervention with a larger sample of Hispanic patients.

The collaborative planning framework that guides the proposed study could be used to modify and prepare other health interventions for vulnerable populations and advance implementation science by developing a systematic approach for helping to close the gap between research and practice in order to reduce health inequities in underserved communities. The adapted intervention will be one of the few to date to focus on a critical public health issue, improving the physical health of Hispanics with SMI and at risk for CVD. In sum, this study blends health-disparities research and implementation science to help reduce the disproportionate burden of medical illness and poor quality of medical care experienced by Hispanics with SMI.

## Competing interests

To LJC, BD, and YW declare that they have no competing interests. RLF received research support from Eli Lilly & Co.

## Authors' contributions

LJC drafted the paper. The other authors reviewed the manuscript and provided extensive feedback. All authors have read and approved the final manuscript.

## Supplementary Material

Additional file 1 **Adapted intervention mapping steps to modify programs to a new patient population and provider group**. The file contains a table describing the adapted intervention mapping steps to modify program to a new patient population and provider group. Click here for file
