# Supplementary material for: Collaborative planning approach to inform the implementation of a healthcare manager intervention for hispanics with serious mental illness: a study protocol
Source: Implement Sci. 2011 Jul 26;6:80. doi: 10.1186/1748-5908-6-80 (PMC3169485; doi:10.1186/1748-5908-6-80)
Supplement: Additional file 1 — Adapted intervention mapping steps to modify programs to a new patient population and provider group. The file contains a table describing the adapted intervention mapping steps to modify program to a new patient population and provider group. [file 1748-5908-6-80-S1.DOC]

**Additional File 1**

| **Step 1: Problem Analysis** |
| --- |
| What new cultural or other population issues are present? |
| What new environmental issues or needs at the interpersonal, organizational, and/or community levels must be considered? |
| What is the organizational and community capacity for the new population and intervention? |
| **Step 2: Review of Intervention Objectives and Theoretical Foundations** |
| **Task 1: Review Behavioral Outcomes** |
| What behavioral outcomes need to be added for the new patient population and provider  group? |
| Which behavioral outcomes may need to be deleted as inappropriate? |
| Which behavioral outcomes may need to be deleted or adapted as impractical? |
| **Task 2: Specify Performance Objectives** |
| Which performance objectives should be deleted as irrelevant? |
| Given the new or revised behavioral outcomes: |
| What performance objectives need to be added? |
| What performance objectives need to be revised? |
| **Task 3: Specify Determinants** |
| What determinants were used in devising the original program? |
| What is the supporting evidence for those determinants? |
| Does the evidence indicate the determinants are relevant to the new population?  Does the evidence indicate the determinants are relevant for the new provider group? |
| Which determinants should be deleted or revised? |
| What determinants need to be added for the new population?  What determinants need to be added for the new provider group? |
| What determinants need to be added for the new performance objectives? |
| **Task 4: Develop Proximal Program Objectives** |
| For each determinant of each performance objective: |
| What are the learning objectives of the existing program? |
| Given the changes in determinants and performance objectives: |
| What learning objectives should be deleted? |
| What learning objectives should be revised? |
| What learning objectives should be added? |
| **Task 5: Identifying Theoretical Foundation** |
| What behavioral theory is associated with each determinant in the original program? |
| Which of these theories is relevant to the new population?  Which of these theories is relevant to the new provider group? |
| Which of these theories is appropriate for the new determinants? |
| What part of the theoretical foundation should be discarded? |
| What should be added to the theoretical foundation of the revised program? |
| **Step 3: Modification of Intervention Methods and Strategies** |
| Are the methods and strategies of the original program effective for the new population? |
| Are the methods and strategies of the original program effective for the new provider  group?  Are they feasible given the new study design? |
| Are they practical given the new community context? |
| Given that methods and strategies are specific to learning objectives: |
| Which methods or strategies should be deleted? |
| Which methods and strategies should be revised? |
| Looking at the new learning objectives and the relevant theories: |
| Which methods can be expanded to cover new learning objectives? |
| What new intervention methods or strategies need to be added? |
| **Step 4: Development of Revised Intervention** |
| Comparing the content of the original intervention with the revised learning objectives: |
| Which learning objectives are well covered by existing content? |
| Which learning objectives are partially addressed by existing content? |
| Which learning objectives require new content? |
| Looking at the program content: |
| What content should remain the same? |
| What content should be adapted or deleted? |
| What content should be added? |
| **Step 5: Development of Adoption and Implementation Plan** |
| Is the new population comparable to that targeted by the original intervention?  Is the new provider group comparable to the provider group used in the original  intervention? |
| If not, how does these differences in population and provider group affect the ability  to replicate the intervention with fidelity and completeness? |
| Does the new community context present new practical or logistical issues? |
| If so, how must adoption and implementation be adapted?  What organizational and community resources can be used to support the adoption and  implementation of the modified intervention?  What organizational and community strategies and methods can be used to support the  adoption and implementation of the modified intervention?  What factors at the individual, interpersonal, organizational, and community level may  impact the sustainability of the modified intervention? |
| **Step 6: Evaluation** |
| What is the evaluation model required for this population and setting? |
| Are the original indicators and measures the most relevant and useful for the new community context, population, provider group, behavioral outcomes, and performance objectives? |
| If not, what new indicators and measures of impact variables are needed? |
| What are the appropriate process measures for this new population, provider group, and  setting? |

Note: Adapted from Tortolero and colleagues (38) and Bartholomew and colleagues (39).
